# Late-onset familial amyloidosis polyneuropathy associated with c.186G>C in transthyretin

**DOI:** 10.31053/1853.0605.v81.n1.40992

**Published:** 2024-03-27

**Authors:** Eugenia Conti, Sebastián Menazzi, Ana Mariel Finkelsteyn, María de Lourdes Figuerola

**Affiliations:** 1 Hospital de Clínicas "José de San Martín" Universidad de Buenos Aires Buenos Aires Argentina

**Keywords:** prealbúmina, amiloidosis familiar, neuropatías amiloides, prealbumin, amyloidosis, familial, amyloid neuropathies, pré-albumina, amiloidose familiar, neuropatias amiloides

## Abstract

**Introduction:**

The most common form of hereditary amyloidosis is associated with variants of transthyretin (*TTR*). Familial amyloidosis polyneuropathy associated with variants of *TTR* (FAP-*TTR*) is an infrequent, multisystemic disease, with predominant involvement of the peripheral nervous system. More than 130 pathogenic variants have been identified so far and most of them are amyloidogenic, being Val30Met the most frequently described

**Case report:**

A 74 year-old male was evaluated for progressive decreased sensitivity and associated loss of strength in four limbs in the previous two years, needing assistance for walking. Areflexia, bilateral tibialis anterior and gastrocnemius atrophy, bilateral anesthesia and apalesthesia were found in lower limbs. Bilateral hypoesthesia was reported in upper limbs. No painful dysesthesia, hyperalgesia or allodynia were found. DNA sequencing of the TTR gene led to the detection of the variant c.186G>C in heterozygous state. The resulting variant (Glu62Asp), located in the critical functional domain, has not been published before

**Conclusion:**

The importance of considering late onset, sporadic FAP-*TTR* as a differential diagnosis of cryptogenic polyneuropathy is highlighted.

## Resumen

**Introducción:**

La forma más común de amiloidosis hereditaria está asociada con variantes de la transtiretina. La polineuropatía amiloidótica familiar asociada con variantes de la *TTR* (FAP-*TTR*) es una enfermedad multisistémica poco frecuente, con afectación predominante del sistema nervioso periférico. Hasta ahora se han identificado más de 130 variantes patogénicas y la mayoría de ellas son amiloidogénicas, siendo Val30Met la más frecuentemente descrita

**Caso clínico:**

Un paciente de 74 años fue evaluado por disminución progresiva de la sensibilidad y pérdida asociada de fuerza en las cuatro extremidades de dos años de evolución, necesitando ayuda para caminar. En las extremidades inferiores se observó arreflexia, atrofia bilateral del tibial anterior y del gastrocnemio, anestesia bilateral y apalestesia. Los miembros superiores presentaban hipoestesia bilateral. No se observaron disestesias dolorosas, hiperalgesia ni alodinia. La secuenciación del ADN del gen *TTR* permitió detectar la variante c.186G>C en estado heterocigoto. La variante resultante (Glu62Asp), localizada en el dominio funcional crítico de la proteína, no ha sido informada con anterioridad

**Conclusión:**

Se destaca la importancia de considerar la FAP-*TTR*esporádica de aparición tardía como un diagnóstico diferencial de la polineuropatía criptogénica.

## Resumo

**Introdução:**

A forma mais comum de amiloidose hereditária está associada às variantes da transtiretina. A polineuropatia amiloidótica familiar associada às variantes *TTR* (FAP-*TTR*) é uma doença multisistêmica rara com envolvimento predominante do sistema nervoso periférico. Mais de 130 variantes patogênicas foram identificadas até agora e a maioria delas são amiloidogênicas, sendo o Val30Met o mais frequentemente descrito

**Relato de caso:**

Um paciente masculino de 74 anos de idade foi avaliado por diminuição progressiva da sensibilidade e perda de força associada em quatro membros nos dois anos anteriores, necessitando de assistência para caminhar. Foram encontradas areflexia, atrofia do tibialis anterior bilateral e o gastrocnemio, anestesia bilateral e apalestesia nos membros inferiores. Hipoestesia bilateral foi relatada em membros superiores. Não foram encontradas disestesia dolorosa, hiperalgesia ou alodinia. A sequenciação do DNA do gene *TTR* levou à detecção da variante c.186G>C em estado heterozigoto. A variante resultante (Glu62Asp), localizada no domínio funcional crítico, não foi publicada anteriormente

**Conclusão:**

A importância de considerar o FAP-TTR esporádico tardio como um diagnóstico diferencial da polineuropatia criptogênica é destacada.

**CONCEPTOS CLAVE**

**¿Qué se sabe sobre el tema?**

La polineuropatía amiloidótica familiar (PAF) asociada a variantes de la transtiretina (gen TTR) es una enfermedad multisistémica. Se han identificado más de 130 variantes patogénicas; la mayoría de ellas son amiloidogénicas, siendo Val30Met la más frecuentemente descrita.

**¿Qué aporta este trabajo?**

Comunicamos el primer caso de PAF-TTR de aparición tardía asociado a la variante c.186G>C. Nuestra afirmación se apoya en las manifestaciones clínicas características, los resultados de las pruebas complementarias y el análisis genético.

**Divulgación**

La amiloidosis familiar es una enfermedad que produce distintas formas de afectación del sistema nervioso periférico (polineuropatía). Su causa es genética y se debe a alteraciones en una proteína llamada transtiretina, como consecuencia de variantes en el gen especifico que la codifica, denominado *TTR*.

Describimos aquí el primer caso relacionado con una variante puntual de dicho gen, remarcando la importancia del diagnóstico precoz de esta enfermedad que, si bien no es curable, puede ser tratada en la actualidad.

## Introduction

The most common form of hereditary amyloidosis is due to variants of transthyretin (*TTR*), ^1^ a protein associated with neurogenesis and nerve regeneration. ^2^ Familial amyloidosis polyneuropathy caused by variants of *TTR*(FAP-*TTR*) is a multisystemic disease with peripheral nervous system involvement and amyloid deposits in the endoneurium. ^3^ FAP-*TTR* variants were first described in endemic areas; currently, FAP-*TTR* is considered a worldwide disease, with a global prevalence of 5000 to 10 000 subjects. ^4^ Nevertheless, prevalence of *TTR*amyloidosis is likely higher than previously recognized. ^5^

Hereditary *TTR* amyloidosis is characterized by autosomal dominant inheritance, due to variants in the *TTR* gene.(4,6-7) More than 130 pathogenic variants have been identified, most of them amyloidogenic, ^6^ with phenotypes including neuropathy, cardiomyopathy, and, infrequently, ocular and cerebromeningeal involvement. ^4^ Val30Met is the most common pathogenic variant. ^8^ A registry has been started to record the significance of variants and phenotypes in hereditary *TTR* amyloidosis. ^9^ Several variants have been linked to clusters of families worldwide, including Thr60Ala, Phe64Leu, Ala97Ser, Glu89Gln, Ser50Arg, Ser77Tyr and Ser77Phe. Other variants have been reported only in a single family. ^8^

We describe the first patient with late-onset amyloidotic polyneuropathy with *TTR*Glu62Asp due to the variant c.186G>C.

## Case report

A 74 year-old male was evaluated for decreased sensitivity in lower limbs during the previous 2 years. Upper limbs were also compromised in the last 12 months. He complained of associated loss of strength in four limbs, needing assistance for walking. Recent constipation and ill-defined class II dyspnea were also added. No orthostatic dizziness was present. He had no personal or family neurological medical history. He was the first of two siblings; his sister died of meningitis when she was 7 months old. His father died at the age of 77 (lung cancer) and his mother at the age of 63 (gallbladder cancer). His parents were non-consanguineous and of Argentinean native origin. He had a 41 year-old healthy son, whose 14 year-old son was diagnosed with autism spectrum disorder, and a 53 year-old healthy daughter, whose two daughters (29 and 15 years old) were healthy.

A neurological examination showed lower limbs areflexia, bilateral anesthesia and apalesthesia; hypotonia, weakness (Medical Research Council Scale grade 3), bilateral tibialis anterior and gastrocnemius atrophy were found in the muscular examination. In addition, bilateral hypoesthesia was reported in upper limbs. No painful dysesthesia, hyperalgesia, thermoalgesic dissociation or allodynia were found. Complete blood count, renal function tests, liver enzymes, glycemia, thyroid function tests, muscle enzymes and lipid profile were within normal range. Urine tests were negative for proteinuria. Test for detection of a serum and urine monoclonal component were negative. Blood tests for rheumatic biomarkers and hepatitis B, hepatitis C and human immunodeficiency virus were also negative.

Other complementary tests results are summarized in Table 1.

**Tabla Nº 1:** Complementary tests

**Specific test**	**Result**
**Four limbs electromyography**	Axonal sensory-motor polyneuropathy with signs of denervation
**Echocardiogram**	Left atrial dilatation, concentric ventricular hypertrophy and preserved ejection fraction
**24-hours electrocardiography (Holter)**	Sinus rhythm with a permanent first-degree atrioventricular block

DNA sequencing of the *TTR*gene was suggested as part of the screening for idiopathic polyneuropathy. DNA samples were collected and amplified using a primers pool designed using the Ion Ampliseq^TM^ software for *TTR*gene, according to the manufacturer recommendations. The analysis was performed by amplicon next generation sequencing using Post Light^TM^ Ion Semiconductor Sequencing in an Ion Personal Genome Machine System^TM^ platform. This methodology led to the detection of the variant c.186G>C in heterozygous state, which is associated with an alteration in the protein direction. The resulting variant (Glu62Asp) is located in the critical functional domain (Peptidase_M14NE-CP-C_like), ^10^ without known benign variants. Variant c.186G>C has not been published before and has been reported only in ClinVar, but not in projects 1000Genomes, Exome Aggregation Consortium, NHLBI and Genome Aggregation Database. A previous report of variant c.186G>T as a cause of *TTR* amyloidosis was found; ^1^ both variants are predicted to produce the same amino acid change. We concluded that this novel variant should be considered likely pathogenic according to the available information, the clinical phenotype suggesting FAP-*TTR* and the ACMG variant classification standards. ^11^ The proband's son and daughter were properly counseled, but they initially refused presymptomatic genetic testing.

## Discussion

We report the first case of late-onset FAP-*TTR*associated with the variant c.186G>C. Our affirmation is supported by the characteristic clinical manifestations, the results of complementary tests and the genetic analysis.

Dupuy *et al* has previously reported a patient with late-onset cardiac amyloidosis with a novel variant (Glu42Asp) in the same nucleotide; ^1^ nevertheless, they described a c.186G>T alteration, while DNA sequencing in our patient showed a previously unreported c.186G>C variant. In both the Dupuy *et al*'s report and our case, the new codon results in transcription of aspartate. However, the case report from Dupuy *et al* presented with isolated late-onset amyloid cardiomyopathy and our patient had a predominant neuropathic phenotype. Phenotypic heterogeneity may be linked to differences among specific pathogenic *TTR* variants, geographic factors, the subtype of endemic versus non-endemic disease, and several probable environmental factors that are currently unknown. Any or all of these factors may be involved in the phenotypic difference of these patients with the same amino acid substitution.

Other *TTR* variants with an amino acid residue substitution at the same codon have been described. Ueno *et al* reported a Japanese 42 year-old male with isolated lower limbs neuropathy and chronic diarrhea. A nerve biopsy showed amyloid deposits and Glu42Gly variant was confirmed by genetic testing. ^12^ The same variant has also been reported by Skare *et al* in four siblings, with a clinical phenotype including peripheral neuropathy, diarrhea, vitreous opacity and cardiomyopathy. ^13^ By contrast, our patient showed polyneuropathy and constipation, without other clinical or echocardiographic alterations.

We underscore the importance of including FAP-*TTR* among early differential diagnosis in patients with presumably idiopathic polyneuropathy. However, a diagnosis delay of 8 years has been reported. ^14^ Misleading diagnoses are related with sporadic, late-onset and varied clinical presentation patterns. ^15^

## Conclusion

Being a currently treatable condition, early diagnosis of FAP-TTR is essential for the rapid introduction of drugs that dramatically improve the quality of life and also for genetic counseling for the patient and at-risk family members. TTR gene sequencing may be considered the gold standard molecular diagnosis test, since this tool improves the probability of detecting new variants, as currently described.
